# Apparent diffusion coefficient values in Modic changes – interobserver reproducibility and relation to Modic type

**DOI:** 10.1186/s12891-022-05610-4

**Published:** 2022-07-22

**Authors:** Magnhild H. Dagestad, Nils Vetti, Per M. Kristoffersen, John-Anker Zwart, Kjersti Storheim, Gunnstein Bakland, Jens I. Brox, Lars Grøvle, Gunn H. Marchand, Erling Andersen, Jörg Assmus, Ansgar Espeland

**Affiliations:** 1 Department of Radiology, Haukeland University Hospital, Jonas Liesvei 65, 5021 Bergen, Norway; 2 Department of Clinical Medicine, University of Bergen, P.O. Box 7804, 5020 Bergen, Norway; 3 Research and Communication Unit for Musculoskeletal Health (FORMI), Division of Neuroscience, Oslo University Hospital HF, Ulleval, Bygg 37b, P.O. Box 4956, Nydalen, 0424 Oslo, Norway; 4 Faculty of Medicine, University of Oslo, P.O. Box 1072 Blindern, 0316 Oslo, Norway; 5 Faculty of Health Science, OsloMet - Oslo Metropolitan University, P.O. Box 4, St. Olavs plass, 0130 Oslo, Norway; 6 Department of Rheumatology, University Hospital of North Norway, P.O. Box 100, 9038 Tromsø, Norway; 7 Department of Physical Medicine and Rehabilitation, Oslo University Hospital HF, P.O. Box 4956 Nydalen, 0424 Oslo, Norway; 8 Department of Rheumatology, Østfold Hospital Trust, P.O. Box 300, 1714 Grålum, Moss, Norway; 9 Departement of Physical Medicine and Rehabilitation, St. Olavs Hospital, Trondheim University Hospital, P.O. Box 3250 Torgarden, 7006 Trondheim, Norway; 10 Departement of Neuromedicine and Movement Science, Norwegian University of Science and Technology (NTNU), 7491 Trondheim, Norway; 11 Department of Clinical Engineering, Haukeland University Hospital, P.O. Box 1400, 5021 Bergen, Norway; 12 Centre for Clinical Research, Haukeland University Hospital, Jonas Liesvei 65, 5021 Bergen, Norway

**Keywords:** MR-imaging, Spine, Imaging sequences, Adults, Skeletal-axial, Observer performance

## Abstract

**Background:**

Modic Changes (MCs) in the vertebral bone marrow were related to back pain in some studies but have uncertain clinical relevance. Diffusion weighted MRI with apparent diffusion coefficient (ADC)-measurements can add information on bone marrow lesions. However, few have studied ADC measurements in MCs. Further studies require reproducible and valid measurements. We expect valid ADC values to be higher in MC type 1 (oedema type) vs type 3 (sclerotic type) vs type 2 (fatty type). Accordingly, the purpose of this study was to evaluate ADC values in MCs for interobserver reproducibility and relation to MC type.

**Methods:**

We used ADC maps (b 50, 400, 800 s/mm^2^) from 1.5 T lumbar spine MRI of 90 chronic low back pain patients with MCs in the AIM (Antibiotics In Modic changes)-study. Two radiologists independently measured ADC in fixed-sized regions of interests. Variables were MC-ADC (ADC in MC), MC-ADC% (0% = vertebral body, 100% = cerebrospinal fluid) and MC-ADC-ratio (MC-ADC divided by vertebral body ADC). We calculated mean difference between observers ± limits of agreement (LoA) at separate endplates. The relation between ADC variables and MC type was assessed using linear mixed-effects models and by calculating the area under the receiver operating characteristic curve (AUC).

**Results:**

The 90 patients (mean age 44 years; 54 women) had 224 MCs Th12-S1 comprising type 1 (*n* = 111), type 2 (*n* = 91) and type 3 MC groups (*n* = 22). All ADC variables had higher predicted mean for type 1 vs 3 vs 2 (*p* < 0.001 to 0.02): MC-ADC (10^− 6^ mm^2^/s) 1201/796/576, MC-ADC% 36/21/14, and MC-ADC-ratio 5.9/4.2/3.1. MC-ADC and MC-ADC% had moderate to high ability to discriminate between the MC type groups (AUC 0.73–0.91). MC-ADC-ratio had low to moderate ability (AUC 0.67–0.85). At L4-S1, widest/narrowest LoA were for MC-ADC 20 ± 407/12 ± 254, MC-ADC% 1.6 ± 18.8/1.4 ± 10.4, and MC-ADC-ratio 0.3 ± 4.3/0.2 ± 3.9. Difference between observers > 50% of their mean value was less frequent for MC-ADC (9% of MCs) vs MC-ADC% and MC-ADC-ratio (17–20%).

**Conclusions:**

The MC-ADC variable (highest mean ADC in the MC) had best interobserver reproducibility, discriminated between MC type groups, and may be used in further research. ADC values differed between MC types as expected from previously reported MC histology.

**Supplementary Information:**

The online version contains supplementary material available at 10.1186/s12891-022-05610-4.

## Background

Diffusion-weighted magnetic resonance imaging (DWI) is based on the random motion or “self-diffusion” of water molecules in a tissue, which depends on its histology [1]. The apparent diffusion coefficient (ADC) is a measurement of the diffusion calculated from the DWI-images [1–3]. Thus, the ADC adds information on function (diffusion) not revealed by imaging of anatomy and histology. DWI was first successfully used to evaluate brain tissue and is now also used in other soft tissue organs, especially for cancer imaging. In the vertebral bone marrow, DWI and ADC measurements are not part of standard clinical imaging protocols but have been applied to study inflammatory and infectious disorders and to differentiate benign from malignant compression fractures [3–9].

Modic changes (MCs) are magnetic resonance imaging (MRI) findings of vertebral bone marrow changes extending from the endplate. They are divided into type 1 (oedema type), 2 (fatty type) and 3 (sclerotic type) based upon T1- and T2-weighted images [10]. Type 1 MCs were related to back pain in some studies [11–13], but the clinical significance of MCs is uncertain [14]. There are limited data on ADC measurements in MCs [4–6, 15], but ADC values have been used to help distinguish type 1 MCs from infectious spondylitis [4, 5] and inflammatory spondyloarthritis [6]. In patients with MCs, DWI represents a research tool and has yet no role in routine imaging. Further research on the relevance of ADC measurements in MCs requires reproducible and valid measurements. We expect valid ADC values to differ according to MC type, since the underlying histology differs [16]. The aim of this study was to evaluate ADC values in MCs for interobserver reproducibility and relation to MC type.

## Methods

This cross-sectional study was based on baseline MRI of 90 consecutive patients aged 25–63 years (mean age 44 years; 54 women) with chronic low back pain and MCs who were included in the Norwegian AIM (Antibiotics In Modic changes)-study, which comprised 180 patients. The current sample size (*n* = 90) was based on a power calculation (see below). Eligibility criteria and methodology of the AIM-study are previously published [17, 18]. In short, all AIM patients had type 1 and/or type 2 MCs, with height ≥ 10% of vertebral body height and diameter > 5 mm, at the level of a previous lumbar disc herniation [17]. The present analysis included any type of MC of that size at any level Th12-S1 with or without disc herniation. Patients with prior low back surgery, except surgery for disc herniation performed more than 1 year earlier, were excluded from the AIM-study. None had lumbar metal implants. All patients gave written informed consent prior to inclusion. The study was approved by the Regional Committees for Medical Research Ethics in South East Norway (ref. no. 2014/158). The current report follows guidelines for reporting reliability and agreement studies [19].

### Images

The patients included in the present analysis underwent MRI of the lumbar spine during the initial phase of AIM from 2015 to 2016 at five centres using identical protocols and 1.5 T scanners (Magnetom Avanto B19, Siemens Healthineers, Erlangen, Germany). This study applied sagittal ADC maps and T1- and T2-weighted non-fat saturated fast spin-echo images (‘T1/T2’). Gradient-echo diffusion weighted echo-planar imaging with fat saturation was performed. The system software generated ADC maps based on b values of 50, 400 and 800 s/mm^2^ (recommended by the vendor) and three orthogonal directions of diffusion sensitization (see protocol details in Table 1).

**Table 1:** DWI with sagittal ADC maps of the lumbar spine

Repetition time (TR)	5500 ms
Echo time (TE)	104 ms
Echo-planar imaging (EPI) factor	192
Number of acquisitions (averages)	3
Number of concatenations	1
Number of slices	17
Matrix (frequency x phase)	192 × 192
Field of view (FoV)	350 mm × 350 mm
Slice thickness	4.0 mm
Interslice gap	0.0 mm
Voxel size	1.8 mm × 1.8 mm × 4.0 mm
Receiver bandwidth	1628 Hz/pixel
Phase encoding direction	Anterior to posterior
Saturation pulses	Anterior, 30 mm
Acquisition time	3 min 48 s
Coverage	From above Th12 to below S2
Phase oversampling	0%
Fat saturation technique	Chemical shift-selective pre-pulse
Parallel acquisition techniques (PAT) mode	None
Distortion correction filters	Yes
b values	50, 400, 800 s/mm^2^
Diffusion weightings (b values)	3
Diffusion encoding scheme	Bipolar

*DWI* Diffusion weighted imaging, *ADC* Apparent diffusion coefficient

Sagittal gradient-echo diffusion weighted echo-planar imaging (EPI) with fat saturation was performed on 1.5 T Magnetom Avanto scanners with B19 software (Siemens Healthineers, Erlangen, Germany). The system software generated ADC maps based on the three tabled b values and an average measure for the three orthogonal directions of diffusion sensitization. The integrated spine array coil was used, and no surface coils

For T1/T2, slice thickness/ interslice gap was 4 mm/0.4 mm, matrix 384 × 269, field of view 300 mm × 300 mm, echo time (ms)/repetition time (ms) 11/575 (T1) and 87/3700 (T2), and echo train length 5 (T1) and 17 (T2). All images were stored and evaluated at a single centre using Agfa Impax 6.5 (Agfa HealthCare, Mortsel, Belgium).

### Evaluation

Two radiologists (A, B), with 6 (A) and more than 10 years of experience (B), independently evaluated levels Th12-S1 (12 endplates) using all sagittal slices. The observers were aware that patients had chronic low back pain but were otherwise blinded to clinical findings. They cross-navigated between the ADC map and the T1/T2 images to ensure ADC was measured in an MC related area. MCs were defined based on T1/T2 images [10, 20] (Table 2). We excluded MCs with height < 10% of vertebral body height or diameter ≤ 5 mm according to one/both radiologists.

**Table 2:** Description of magnetic resonance imaging variables

MCs	Signal changes in the vertebral bone marrow that extend from the endplate. Excluded are changes separated from the endplate, abutting the endplate with a smaller base than height, or extending through the endplate (Schmorl’s hernias).
MC type	MC type 1 is hypointense on T1- and hyperintense on T2-weigted images. Type 2 is hyperintense on T1 and hyper- or isointense on T2. Type 3 is hypointense on T1 and T2. Borderline type 1 vs 2 with near isointense T1 signal is rated as type 2.
MC-ADC	Highest mean ADC value in a 41.8 mm^2^ ROI in the vertebral body marrow at the endplate with MC on T1/T2. The ROI is placed in the most intense MC related region on the ADC map. If the MC region has uniform intensity on the ADC map, the ROI is placed in the MC area with largest height on T1/T2. ADC is not measured for MCs with height < 10% of vertebral body height or diameter ≤ 5 mm. A ROI of only 41.8 mm^2^ (diameter 7 mm) is used to accommodate small MCs.
CSF-ADC	Mean ADC value in the CSF in a 41.8 mm^2^ ROI at the level of the MC affected vertebral unit, measured in the midsagittal image, or the next image left or right, avoiding non-CSF structures (like nerve roots seen on T1/T2). CSF-ADC is measured in an area with uniform intensity and no pulsation artefacts, behind the lower half of the cranial vertebra of the vertebral unit (e.g., behind L3 if the MC is superior or inferior to the L3/L4 disc) if possible, and otherwise behind the caudal vertebra of the unit or at the next vertebral unit caudally or cranially.
Body-ADC	Mean ADC value in a 94 mm^2^ ROI in normal (on T1/T2) vertebral body marrow near the MC. The ROI is placed close to the endplate in the central anteroposterior third of the normal opposite part (caudally or cranially) of the vertebral body with the MC. If the opposite part is not normal, and always when the MC is in S1, the ROI is placed in the nearest vertebra above, in its caudal part if possible, and otherwise in its cranial part. The measurement is first considered in the midsagittal image and then, if necessary, considered in the next image (left or right) before a new location may be considered. The larger 94 mm^2^ ROI is used to average more pixels without including the central vertebral vein in the ROI.
MC-ADC-ratio	Calculated as MC-ADC / Body-ADC
MC-ADC%	Calculated as (MC-ADC – Body-ADC) × 100% / (CSF-ADC – Body-ADC)

*MC* Modic change, *ADC* Apparent diffusion coefficient, *ROI* Region of interest, *CSF* Cerebrospinal fluid

**Fig. 1:**
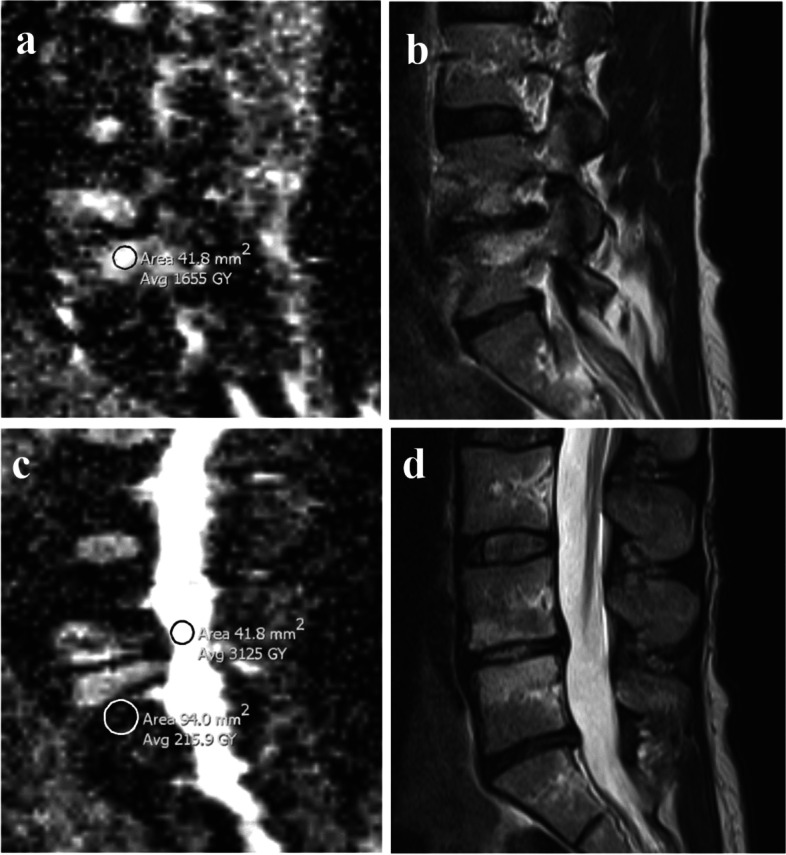
Measurements of ADC values. (**a**-**d**) A 50-year-old woman with chronic low back pain. ADC maps (**a**, **c**) and corresponding T2 weighted fast spin echo images (**b**, **d**) showing MCs at the L4/L5 level. ADC measurements (Avg GY corresponding to mean 10^−6^ mm^2^/s) included (**a**) highest mean ADC value in the MC region (1655 in a 41.8 mm^2^ ROI) and (**c**) mean ADC in normal vertebral body marrow (215.9 in a 94 mm^2^ ROI) and in CSF (3125 in a 41.8 mm^2^ ROI). Midsagittal images were used for measurements in CSF at the level of the MC and close to the endplate in normal vertebral body marrow near the MC. ADC, apparent diffusion coefficient. MC, Modic change. ROI, region of interest. CSF, cerebrospinal fluid

For each MC, ADC was measured in the MC related area that was most intense on the ADC map, in normal vertebral body marrow, and in cerebrospinal fluid (CSF) using a circular region of interest (ROI) with predefined size (Fig. 1, Table 2). To limit variation in ADC measurements, we did not use freely shaped ROIs. If the MC area had uniform intensity on the ADC map, ADC was measured in the area where the MC had largest height on T1/T2.

The following ADC variables were analysed (Table 2): (a) the MC related ADC value (10^− 6^ mm^2^/s) (MC-ADC), (b) MC-ADC in percent (MC-ADC%) where 0% = ADC in normal vertebral body marrow and 100% = ADC in CSF, and (c) MC-ADC divided by the vertebral body ADC (MC-ADC-ratio).

Prior to any ADC measurements, as part of a previous study [20], MC type was independently assessed by three radiologists (B, C, D), each with more than 10 years’ spine MRI experience. MCs were classified in types 1, 2 and 3 [10] (Table 2).

Mixed MC types were classified as primary (most extensive) type / and secondary type, i.e., MC types 1/2, 1/3, 2/1, 3/1, 2/3 and 3/2. Finally, in the present study, MCs were grouped into a type 1 group (any MC containing type 1), type 2 group (pure type 2 MCs) or type 3 group (MC types 3, 3/2, and 2/3).

Conclusive MC type was based on the agreement of at least two of the three observers B, C, and D. If all three disagreed, MC type was decided in consensus with observer A. The conclusive value for ADC variables was the mean of the values reported by observers A and B. The height of the MC into the vertebral body was measured in mm in our previous study [20] and is reported here as the mean of the values reported by observers C and D.

### Pilot study

Prior to this study, observers A, B, and C performed a pilot study on 10 patients, to determine the ADC evaluation criteria and align the measurements. The pilot study patients were not included in the present study.

### Hypothesis

A priori, we hypothesized that ADC values were higher in the type 1 MC group vs the type 3 group and higher in the type 3 group vs the pure type 2 group. Rationale: Compared to type 2 and 3, type 1 MCs are likely to contain more inflammatory oedema, favouring motion of water molecules and increasing the ADC value. Trabecular thickening / sclerosis restricts water motion, and less trabecular thickening in type 1 vs type 3 MCs [21] also suggests higher ADC values in type 1. The large hydrophobic fatty cells in type 2 MCs may restrict water motion / reduce ADC values more than does the fibrovascular granulation tissue with inflammatory cells in type 1 MCs [10, 16] and the trabecular thickening in type 3 MCs [21]. In an MC containing type 1 but also type 2 and/or 3, we expected type 1 to contribute the highest ADC value.

### Statistical analyses

The reproducibility analysis was restricted to MCs extending from one of the four lowest endplates (L4-S1), because of low prevalence (< 10%) of MCs at the other endplates [22]. Interobserver reliability at each endplate was assessed by Cohen’s kappa (MC presence and type) and intraclass correlation coefficients (ICCs) (ADC variables). We used 2-way random effects, absolute-agreement, average-measures ICCs. ADC variables were also analyzed using Bland Altman plots with mean of differences ±1.96 SD (limits of agreement, LoA) at each endplate and pooled across all four endplates L4-S1. We further calculated the proportion of differences exceeding 50% of the observers’ mean value for each ADC variable across L4-S1. We used 50% as cut-off because LoA were 5% ± 45% for ADC in vertebral bone marrow in a prior intra rater study [23]. Interpretation of Cohen’s kappa: 0.00–0.20 poor; 0.21–0.40 fair; 0.41–0.60 moderate; 0.61–0.80 good; 0.81–1.00 very good reliability [22]. ICC values were regarded to indicate poor (< 0.50), moderate (0.50–0.75), good (0.76–0.90) or excellent (> 0.90) reliability [24].

The relation between each ADC variable and MC type group was analysed using conclusive ADC values from MCs extending from one of the 12 endplates Th12-S1. Linear mixed-effects models were conducted using the ADC variable as dependent variable, MC type group as fixed effect, and endplate and patient as random effects. In each MC type group, the model returned a predicted mean value of the ADC variable that had been adjusted for data dependency between MCs at different endplates within the same patient. We also assessed the ability of each ADC variable to discriminate between the MC type groups by calculating the area under the receiver operating characteristic curve (AUC). We graded the discriminatory ability as low (AUC 0.5 to < 0.7), moderate (0.7 to < 0.9), or high (0.9–1.0) [25].

Mixed-effect models were conducted in R 4.0 (R Foundation, Vienna, Austria), using normality plots of standardized residuals and fitted values to assess model assumptions. All other analyses were performed using MedCalc 17.6 (MedCalc Software, Ostend, Belgium). Plots were made using Matlab 9.5 (Mathworks, Massachusetts, United States) and MedCalc 17.6. The significance level was 0.05.

### Sample size

Previously reported ADC values (recalculated to 10^− 6^ mm^2^/s) were 624–1800 (SD 120–316) in type 1 MCs [4, 5] and 500 (SD 160) in type 2 MCs [4]. Assuming SD 300 for ADC in both of two MC groups, 36 MCs in each group are sufficient to detect a mean ADC difference of 200 between the groups (β = 0.2, two-sided α = 0.05). We needed 31 MCs at a given endplate to get a precision of ±0.10 (95% confidence level) for an ICC of 0.85. We expected 90 patients to have enough MCs to compare the ADC variables between the three MC type groups and to estimate their reliability.

## Results

We included MCs from all 90 patients, 224 MCs in total (Table 3). These were 111 type 1 group MCs (any type 1), 91 type 2 group MCs (pure type 2), and 22 type 3 group MCs (20 type 2/3, 2 type 3/2, 0 type 3). MC height was mean 10.7 mm (SD 3.6 mm) and was ≥7 mm in 85% of the MCs (191/224). For reproducibility analyses, 201 MCs at L4-S1 were included.

**Table 3:** Distribution of Modic types across the lumbar spine

Modic type	Th12/L1	L1/L2	L2/L3	L3/L4	L4/L5	L5/S1	Total
Pure 1	0	0	0	1	3	11	15 (7)
1/2	0	0	2	4	19	20	45 (20)
1/3	0	0	0	0	7	7	14 (6)
2/1	0	0	3	0	8	25	36 (16)
3/1	0	0	1	0	0	0	1 (0.4)
Pure 2	2	2	4	3	35	45	91 (41)
2/3	0	0	1	0	7	12	20 (9)
3/2	0	0	0	0	0	2	2 (0.9)
Pure 3	0	0	0	0	0	0	0
Total	2 (0.9)	2 (0.9	11 (5)	8 (4)	79 (35)	122 (54)	224 (100)

Tabled values are numbers (%)

### Interobserver reproducibility

The interobserver reliability was very good (kappa 0.85–0.96) for MC presence but varied from moderate to very good (kappa 0.41–0.81) for MC type group (Additional file 1**,** Table A1) and good to excellent (ICC 0.84–0.98) for the three ADC variables (Table 4).

**Table 4:** Interobserver reliability for ADC variables

	L4/L5 superior to disc, *n* = 40	L4/L5 inferior to disc, *n* = 39	L5/S1 superior to disc, *n* = 62	L5/S1 inferior to disc, *n* = 60
MC-ADC	0.97 (0.94–0.98)	0.95 (0.90–0.97)	0.97 (0.94–0.98)	0.98 (0.97–0.99)
MC-ADC%	0.94 (0.86–0.97)	0.91 (0.84–0.96)	0.96 (0.94–0.98)	0.97 (0.95–0.98)
MC-ADC-ratio	0.86 (0.74–0.92)	0.87 (0.75–0.93)	0.84 (0.75–0.91)	0.84 (0.73–0.90)

*ADC* Apparent diffusion coefficient, *MC* Modic change. MC-ADC, ADC in MC. MC-ADC%, ADC in MC in percent (0% = vertebral body, 100% = cerebrospinal fluid). MC-ADC-ratio, ADC in MC divided by ADC in normal vertebral body marrow

Values are intraclass correlation coefficients (95% confidence intervals)

**Fig. 2:**
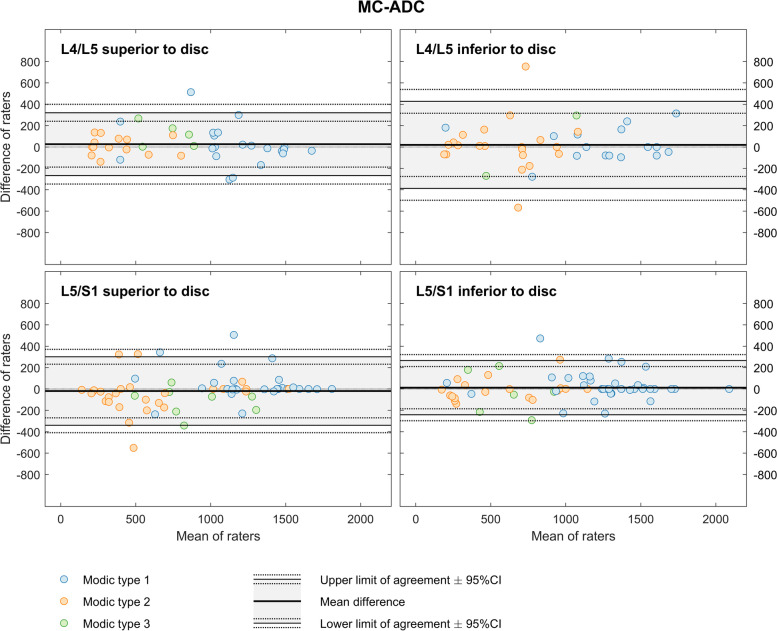
Bland-Altman plots for MC-ADC. The figure shows results for two radiologists who measured MC-ADC in a total of 201 MCs at the four endplates L4-S1. MC, Modic change. ADC, apparent diffusion coefficient. MC-ADC, ADC in MC

For MC-ADC, values (10^− 6^ mm^2^/s) from both observers ranged from 108 to 2029 (mean 913) across the 201 MCs L4-S1. Widest LoA were 20 ± 407 (at L4-L5 inferior to disc) and narrowest LoA were 12 ± 254 (at L5/S1 inferior to disc) (Fig. 2).

**Fig. 3:**
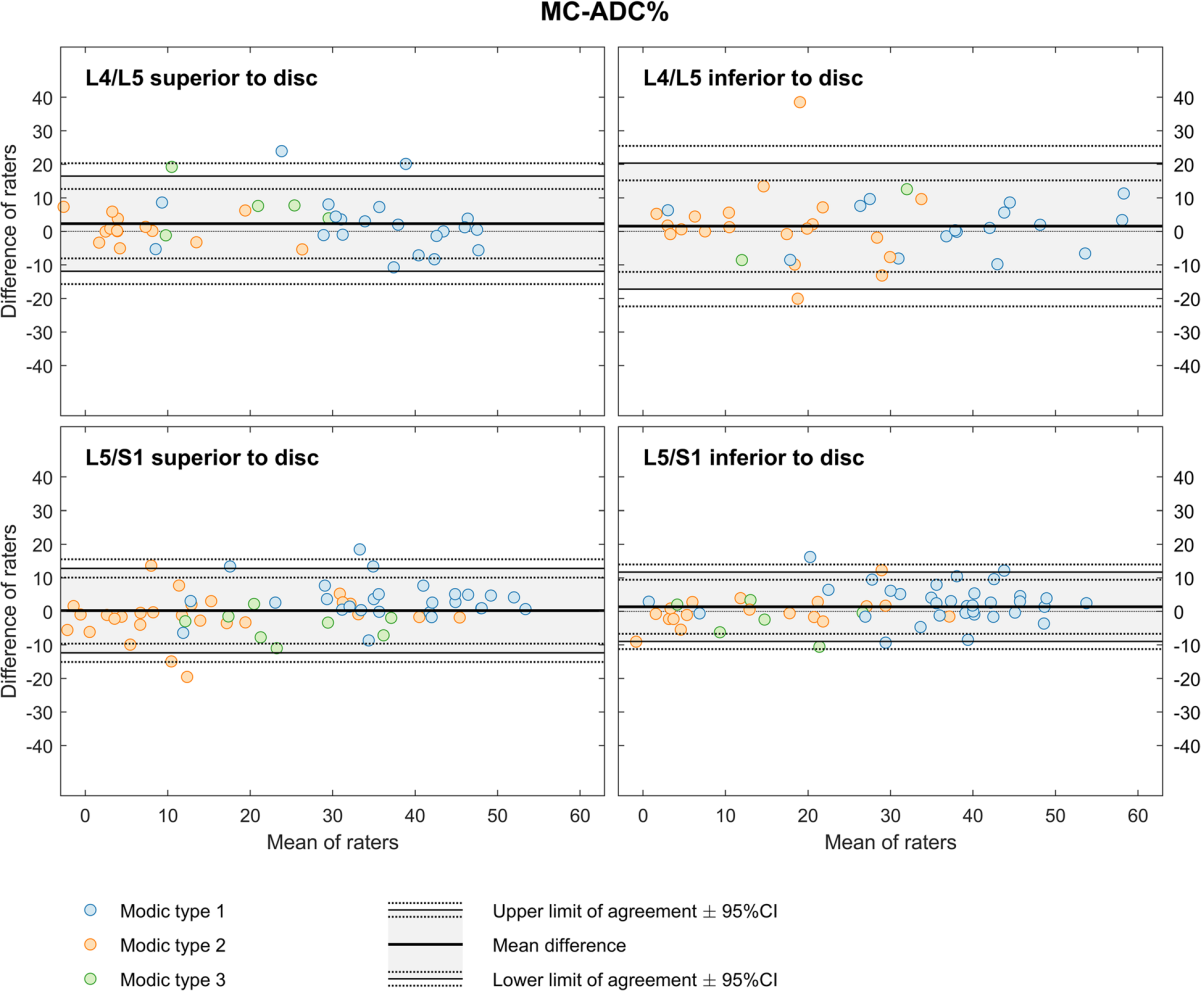
Bland-Altman plots for MC-ADC%. The figure shows results for two radiologists who measured MC-ADC% in a total of 201 MCs at the four endplates L4-S1. MC, Modic change. ADC, apparent diffusion coefficient. MC-ADC%, ADC in MC in percent (0% = vertebral body, 100% = cerebrospinal fluid)

MC-ADC% ranged from 6 to 76 (mean 25.6) and had widest and narrowest LoA of 1.6 ± 18.8 and 1.4 ± 10.4 (Fig. 3).

**Fig. 4:**
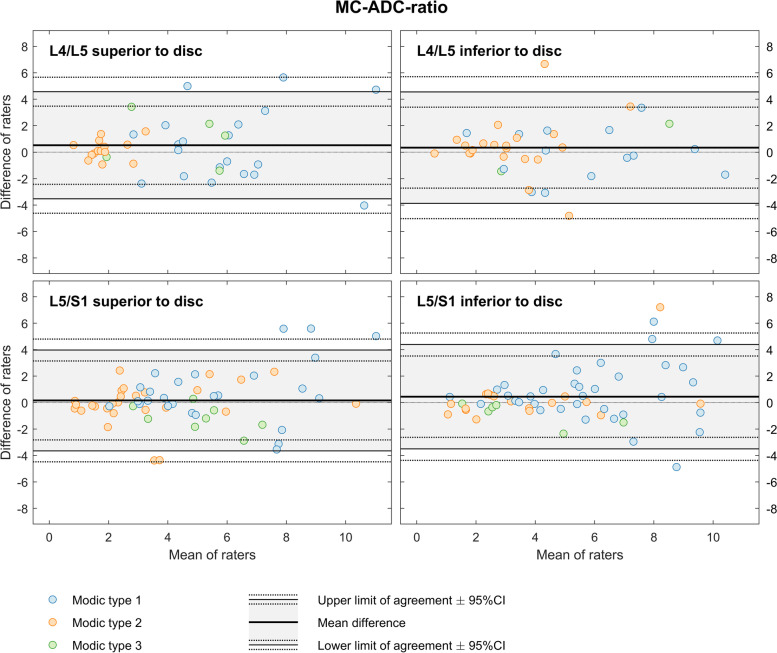
Bland-Altman plots for MC-ADC-ratio. The figure shows results for two radiologists who measured MC-ADC-ratio in a total of 201 MCs at the four endplates L4-S1. MC, Modic change. ADC, apparent diffusion coefficient. MC-ADC-ratio, ADC in MC divided by ADC in normal vertebral body marrow

MC-ADC-ratio ranged from 0.5 to 15.6 (mean 4.7) with widest and narrowest LoA 0.3 ± 4.3 and 0.2 ± 3.9 (Fig. 4).

Pooled LoA across L4-S1 were for MC-ADC (10^− 6^ mm^2^/s) 7 ± 316, MC-ADC% 1.2 ± 13.8, and MC-ADC-ratio 0.4 ± 4.0. The upper border of these LoA reached 35, 59, and 94% of the mean value for MC-ADC (913), MC-ADC% (25.6) and MC-ADC-ratio (4.7), respectively, across all 201 MCs L4-S1.

The difference between the two observers was > 50% of their pairwise mean in 18 (9%) of the 201 MCs for MC-ADC, 41 MCs (20%) for MC-ADC%, and 34 MCs (17%) for MC-ADC-ratio.

Reproducibility parameters for ADC values in CSF and normal vertebral body marrow are shown in Additional file 1, Table A2.

### ADC values by MC type group

Unadjusted mean values of the three MC related ADC variables are shown in Table 5.

**Table 5:** Unadjusted mean for ADC variables by Modic type group

Modic group	Number of MCs	MC-ADC	MC-ADC%	MC-ADC-ratio
**Type 1**	111	1226 (352)	36.3 (12.2)	6.0 (2.5)
**Type 2**	91	535 (306)	12.7 (11.1)	3.0 (1.9)
**Type 3**	22	786 (290)	21.1 (9.8)	4.5 (1.9)

*ADC* Apparent diffusion coefficient, *MC* Modic change. MC-ADC, ADC in MC. MC-ADC%, ADC in MC in percent (0% = vertebral body, 100% = cerebrospinal fluid). MC-ADC-ratio, ADC in MC divided by ADC in normal vertebral body marrow

Tabled are mean values (standard deviation) across Th12-S1 in each of three Modic type groups including a total of 224 MCs in 90 patients

**Fig. 5:**
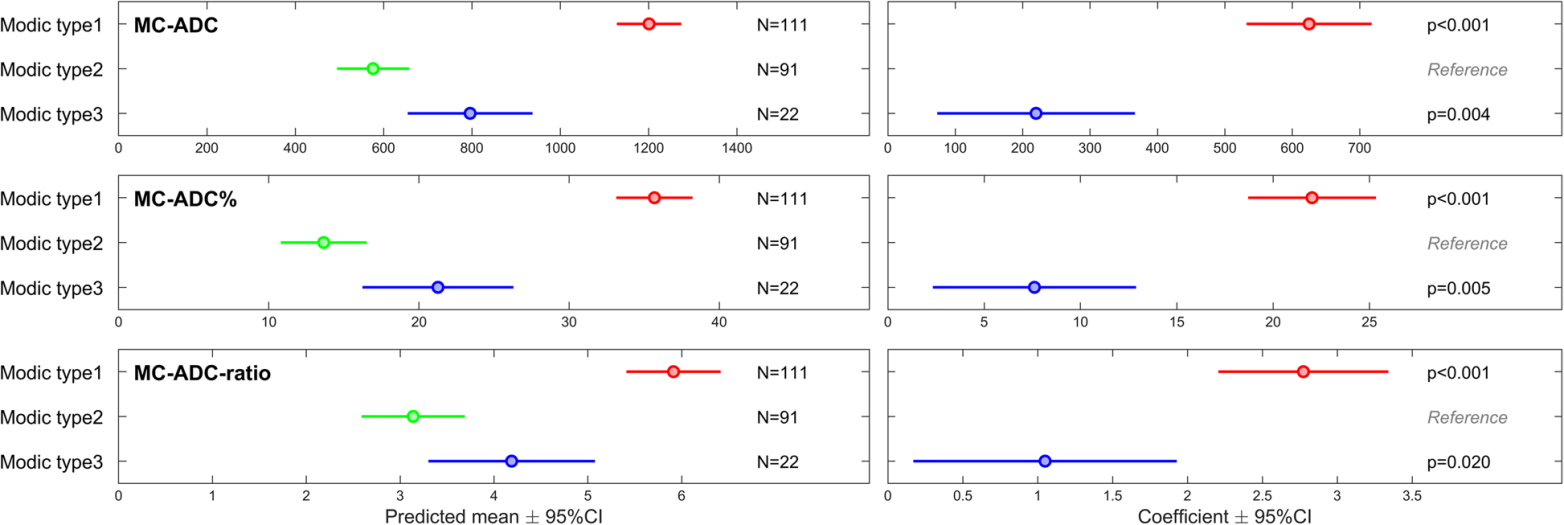
ADC variables according to Modic type group. The left panel shows predicted means from linear mixed-effects analyses for three ADC variables in each of three Modic type groups including a total of 224 MCs Th12-S1 in 90 patients. The right panel shows regression coefficient for Modic type 1 and type 3 groups using type 2 group as reference. ADC, apparent diffusion coefficient. MC, Modic change. MC-ADC, ADC in MC. MC-ADC%, ADC in MC in percent (0% = vertebral body, 100% = cerebrospinal fluid). MC-ADC-ratio, ADC in MC divided by ADC in normal vertebral body marrow

Adjusted for data dependency within patients in the linear mixed-effects models, the predicted means for the ADC variables were higher in the type 1 vs type 3 MC group and in the type 3 vs type 2 MC group (*p* ≤ 0.001 to 0.02) (Fig. 5).

Predicted mean for type 1 vs 3 vs 2 was for MC-ADC (10^− 6^ mm^2^/s) 1201 vs 796 vs 576, for MC-ADC% 36 vs 21 vs 14, and for MC-ADC-ratio 5.9 vs 4.2 vs 3.1.

**Fig. 6:**
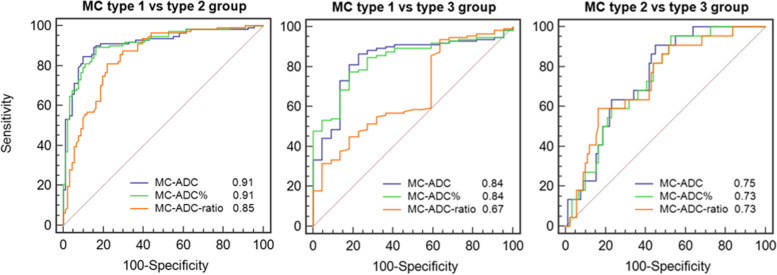
Ability of ADC variables to discriminate between Modic type groups. The figure shows receiver operating characteristic curves and AUC values describing the ability of each ADC variable to discriminate between the Modic type groups for 224 MCs Th12-S1 in 90 patients. MC-ADC and MC-ADC% discriminated better between MC type 1 and type 2, and between type 1 and type 3 than did MC-ADC-ratio (p 0.005 to < 0.001). The ability to discriminate between type 3 and type 2 did not differ between the three variables. ADC, apparent diffusion coefficient. MC, Modic change. AUC, area under the curve. MC-ADC, ADC in MC. MC-ADC%, ADC in MC in percent (0% = vertebral body, 100% = cerebrospinal fluid). MC-ADC-ratio, ADC in MC divided by ADC in normal vertebral body marrow

The ability to discriminate between the MC type groups was moderate to high for MC-ADC and MC-ADC% (AUC 0.73–0.91) and low to moderate for MC-ADC-ratio (AUC 0.67–0.85) (Fig. 6).

Supplementary ADC data are found in Additional file 1, Table A3 and Fig. A1.

## Discussion

This study provides new data on interobserver reproducibility for ADC values in MCs. We found relatively better reproducibility for MC-ADC than for MC-ADC% and MC-ADC-ratio. To our knowledge, this is also the first study to show higher ADC values for a type 1 vs a type 3 MC group and for a type 3 vs a pure type 2 MC group, supporting our hypothesis based on histology of MCs [10, 16, 21].

We tested the hypothesis of ADC differences between MC types to assess the construct validity of the ADC variables [26, 27]. ADC maps cannot replace images used to discriminate between MC types. The discriminative ability still supports the validity of MC-ADC and MC-ADC% and weakens the validity of MC-ADC-ratio. To evaluate how well ADC values represent actual diffusion (criterion validity), one could perform DWI of phantoms with defined diffusion characteristics [28–30].

Pooled LoA suggested that 95% of differences in MC-ADC between observers can be expected to fall within 7 ± 316 (10^− 6^ mm^2^/s). This is relevant when different observers measure MC-ADC in the same patient. ICC ≥ 0.95 indicated that MC-ADC distinguished well between the patients [31], despite LoA reached 35% of the mean across L4-S1. The ICC quantifies the between-subject variability in relation to the measurement error [31, 32]. Thus, the high ICC values (≥ 0.95) may reflect the large variability in MC-ADC values between the patients in our sample. In more homogenous samples the ICC will be lower.

No previous study has reported specifically on the reproducibility of ADC values in MCs. A study of ADC measurements in active spondyloarthritis foci and type 1 MCs [6] reported interobserver ICCs of 0.89–0.98. Other studies on ADC values in bone marrow lacked interobserver data [23, 33]. Our LoA for ADC in normal lumbar bone marrow (4% ± 56%) (Additional file 1, Table A2) were only slightly wider than previously reported for intra observer LoA (5% ± 45%) [23]. ADC values have been found to be less reproducible in bone marrow than in soft tissues [23]. Thus, our results seem to agree with relevant prior studies.

Standardized ROIs, pilot testing, and clear instructions for where to measure probably reduced the variability of the ADC measurements. MC-ADC implied a single measurement, avoiding variation from measurements in CSF and normal bone marrow. This may partly explain the relatively better reproducibility for MC-ADC compared to MC-ADC% and MC-ADC-ratio. We included the two latter variables since it had been found useful in prior studies of ADC values to standardize lesion values against normal tissue values [34–36]. However, in our study this approach added variability. Compared to MC-ADC, MC-ADC-ratio also discriminated less well between the MC type groups. MC-ADC seems more feasible, reproducible, and promising for use in further research.

In line with our results, Belykh et al. found higher mean ADC (10^− 6^ mm^2^/s) in type 1 vs type 2 MCs (498 vs 223, *p* < 0.001) [15]. Prior statistical comparisons of ADC values between all three MC type groups are lacking. In a study with 20 MCs, mean ADC (recalculated to 10^− 6^ mm^2^/s) was descriptively reported to be 624, 500, and 756 in type 1, 2, and 3 MCs, respectively [4]. Thus, ADC values differed between studies. Our mean ADC value of 1226 in type 1 MCs was midways in the range of previous values (498 to 1800) [4, 5, 15], and close to what was found in spondyloarthritis foci (1240) [36].

Many factors can affect ADC values in MCs, such as MRI technique (sequence parameters, b values, fat suppression) [37–40], ROI size and location, type of ADC measure (mean, percentile, histogram), and the definition of MC type (e.g., pure, mixed). Lack of information on mixed MC types, ROI size, and exact location of the ROI in the MC further complicates a comparison of the ADC values [5, 15].

### Strengths and limitations

Strengths of this study are standardized MRI methodology, well-defined criteria for measuring ADC, and a large enough sample size to compare ADC values between MC type groups. A limitation is that our type 3 MC group was dominated by type 2/3 MCs, which may have reduced its ADC values. Partial volume effect can bias ADC measurements in MCs. This was likely a minor issue in our study, since 85% of the MCs appeared clearly larger (based on height ≥ 7 mm) than the slice thickness applied (4 mm) and at least as large as the ROI used (diameter 7 mm). The interobserver reliability for MC type group varied, reflecting difficulties in assessing signal intensities in MCs, especially in mixed MC types, which were prevalent (Table 3). The observers were experienced and had performed a pilot study. Interobserver differences may be larger between less experienced radiologists. We did not assess intra observer agreement, which is often better than the interobserver agreement [41–44].

The ADC maps showed some noise and distortion (Fig. 1), which are common problems in spine DWI [40]. The single-shot echo-planar imaging method applied is prone to susceptibility artefacts, which can influence ADC values. The DWI sequence (3 min 48 s) was part of an extensive MRI protocol where each sequence had been shortened to reduce total scan time and make the protocol feasible at all study centres. Longer acquisition time could have been used to improve the ADC maps [45, 46]. New DWI methods like RESOLVE (readout segmentation of long variable echo-trains), can also provide better image quality but were not available to us at the time [47]. The DWI method we used should be possible to apply at most MRI centres. Importantly, we used T1/T2 images as anatomical references when measuring ADC, and the modest quality of the ADC maps hardly affected the overall results.

### Implications

Our findings have some implications for future research. Firstly, MC-ADC may be preferable when all study participants undergo identical DWI protocols. Secondly, the intra observer repeatability of the ADC variables and their reproducibility with other and improved DWI protocols should be clarified. Finally, the clinical relevance of measuring ADC in MCs is unknown and should be investigated, especially in the most inflammatory type 1 MC group. In inflammatory lesions of spondyloarthritis and sacroiliitis, ADC measures were related to disease activity [36, 48].

## Conclusions

ADC values of MCs had overall moderate interobserver reproducibility and they differed between MC types as hypothesized. The reproducibility was best for MC-ADC - measured in a ROI of predefined size - without standardization against normal bone marrow or CSF. This variable appears feasible, reliable, and valid to use in further research.

## Supplementary Information

**Additional file 1.**

**Additional file 2.**

**Additional file 3.**

## Data Availability

Requests to access data should be addressed to kjersti.storheim@medisin.uio.no. De-identified individual participant data will be available to medical researchers by request in accordance with local legislation and ethical approval. All proposals requesting data access will need to specify an analysis plan and will need approval of the scientific board before any data can be released.

## Abbreviations

ADC: Apparent diffusion coefficient
AUC: Area under the curve
CSF: Cerebrospinal fluid
CI: Confidence interval
DWI: Diffusion weighted imaging
LoA: Limits of agreement
MCs: Modic Changes
MRI: Magnetic resonance imaging
ROI: Region of interest
SD: Standard deviation
T1/T2: T1- and T2-weighted non-fat saturated fast spin-echo images
