# Supplementary material for: Apparent diffusion coefficient values in Modic changes – interobserver reproducibility and relation to Modic type
Source: BMC Musculoskelet Disord. 2022 Jul 22;23:695. doi: 10.1186/s12891-022-05610-4 (PMC9306145; doi:10.1186/s12891-022-05610-4)

Appendix

# **Apparent diffusion coefficient values in Modic changes – interobserver reproducibility and relation to Modic type**

# **Table A1. Interobserver reliability for Modic changes (MCs) in 90 patients**

| Observers | L4/L5 superior to disc | L4/L5 inferior to disc | L5/S1 superior to disc | L5/S1 inferior to disc |
| --- | --- | --- | --- | --- |
| MC present, yes/no | | | | |
| A *vs* B | 0.96 (0.89 – 1.00) | 0.93 (0.85 – 1.00) | 0.89 (0.79 – 1.00) | 0.85 (0.72 – 0.96) |
| MC type group, 1/2/3 | | | | |
| B *vs* C | 0.78 (0.62 – 0.95) | 0.81 (0.64 – 0.98) | 0.53 (0.36 – 0.69) | 0.60 (0.42 – 0.77) |
| C *vs* D | 0.69 (0.49 – 0.89) | 0.43 (0.23 – 0.63) | 0.57 (0.41 – 0.74) | 0.59 (0.43 – 0.76) |
| B *vs* D | 0.58 (0.37 – 0.79) | 0.41 (0.22 – 0.59) | 0.52 (0.35 – 0.68) | 0.58 (0.42 – 0.75) |
| Values are unweighted Cohen’s kappa (95% confidence interval) for presence (yes/no) of MCs with height ≥ 10% of vertebral body height and diameter > 5 mm, and for MC type group 1 (any type 1) *vs* 2 (pure type 2) *vs* 3 (types 3, 3/2, 2/3). | | | | |

# **Table A2. Interobserver reproducibility for Body-ADC and CSF-ADC**

|  | L4/L5 superior to disc, n = 40 | L4/L5 inferior to disc, n = 39 | L5/S1 superior to disc, n = 62 | | L5/S1 inferior to disc, n = 60 |
| --- | --- | --- | --- | --- | --- |
| Body-ADC, ICC | 0.67 (0.38 – 0.82) | 0.68 (0.39 – 0.83) | 0.88 (0.81 – 0.93) | | 0.90 (0.82 – 0.94) |
| CSF-ADC, ICC | 0.66 (0.32 – 0.82) | 0.65 (0.30 – 0.82) | 0.63 (0.35 – 0.78) | | 0.58 (0.26 – 0.75) |
| Body-ADC, LoA | 15 ± 143 | 4 ± 178 | 10 ± 121 | | 11 ± 118 |
| CSF-ADC, LoA | 114 ± 425 | 109 ± 422 | 64 ± 260 | | 67 ± 262 |
| Body-ADC, LoA% (based on mean of Body-ADC values across L4-S1) | | | | 4% ± 56% | |
| Body, normal vertebral body marrow. ADC, apparent diffusion coefficient. CSF, cerebrospinal fluid. ICC, intraclass correlation coefficient (with 95% confidence interval). LoA, limits of agreement.  Column headings show endplate with Modic change. Two radiologists (A, B) independently measured Body-ADC at a different endplate near the Modic change and CSF-ADC at the Modic change level.  ADC values (10^-6^ mm^2^/s) from both observers had range (mean, standard deviation) 64 to 702 (214, 92) for Body-ADC and 2031 to 3374 (2955, 183) for CSF-ADC. | | | | | |

# **Table A3. Unadjusted mean values for ADC variables by Modic type**

|  |  | **MC-ADC** | | | **MC-ADC%** | | | **MC-ADC-ratio** | | |
| --- | --- | --- | --- | --- | --- | --- | --- | --- | --- | --- |
| **Modic type** | **n** | **A** | **B** | **Mean** | **A** | **B** | **Mean** | **A** | **B** | **Mean** |
| **Pure 1** | 15 | 1281 | 1307 | 1294 | 36.1 | 39.7 | 37.9 | 5.0 | 6.1 | 5.7 |
| **1/2 & 1/3** | 59 | 1289 | 1325 | 1307 | 37.6 | 40.1 | 38.8 | 5.8 | 6.5 | 6.1 |
| **2/1 & 3/1** | 37 | 1059 | 1076 | 1067 | 30.4 | 32.7 | 31.6 | 5.7 | 6.2 | 6.0 |
| **All type 1** | 111 |  |  | 1226 |  |  | 36.3 |  |  | 6.0 |
| **Pure 2** | 91 | 544 | 526 | 535 | 12.5 | 12.9 | 12.7 | 2.9 | 3.2 | 3.1 |
| **2/3 & 3/2** | 22 | 798 | 775 | 786 | 21.2 | 21.0 | 21.1 | 4.7 | 4.4 | 4.5 |
| ADC, apparent diffusion coefficient (10^-6^ mm^2^/s). MC, Modic change. MC-ADC, ADC in MC. MC-ADC%, ADC in MC in percent (0% = vertebral body, 100% = cerebrospinal fluid). MC-ADC-ratio, ADC in MC divided by ADC in normal vertebral body marrow.  Two radiologists (A, B) independently measured ADC variables for 224 MCs Th12-S1 in 90 patients. | | | | | | | | | | |

# **Figure A1. Distribution of residuals in the linear mixed-effects analyses**


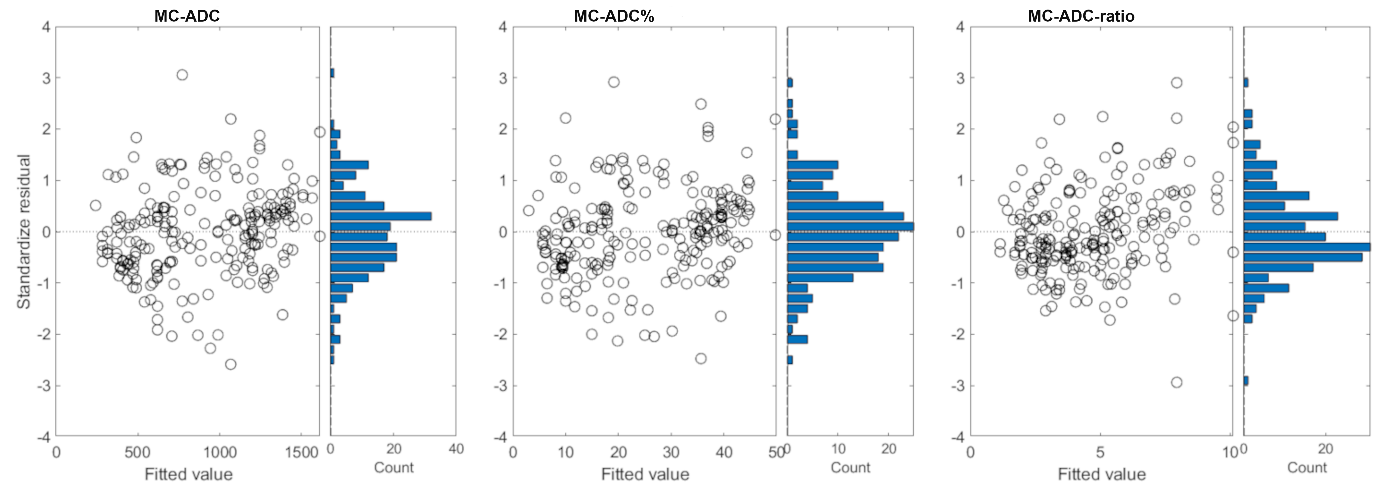

Supplement: Supplementary file 1 — Additional file 1. [file 12891_2022_5610_MOESM1_ESM.docx]
