# Supplementary material for: Apparent diffusion coefficient values in Modic changes – interobserver reproducibility and relation to Modic type
Source: BMC Musculoskelet Disord. 2022 Jul 22;23:695. doi: 10.1186/s12891-022-05610-4 (PMC9306145; doi:10.1186/s12891-022-05610-4)
Supplement: Supplementary file 3 — Additional file 3. [file 12891_2022_5610_MOESM3_ESM.docx]

GRRAS checklist for reporting of studies of reliability and agreement

Version based on Table I in: Kottner J, Audigé L, Brorson S, Donner A, Gajeweski BJ, Hróbjartsson A, Robersts C, Shoukri M, Streiner DL. Guidelines for reporting reliability and agreement studies (GRRAS) were proposed. J Clin Epidemiol. 2011;64(1):96-106

2

| **Section** | **Item #** | **Checklist item** | **Reported on page #** |
| --- | --- | --- | --- |
| Title/Abstract | 1 | Identify in title or abstract that interrater/intrarater  reliability or agreement was investigated. | 1-4 |
| Introduction | 2 | Name and describe the diagnostic or measurement device of interest explicitly. | 4 |
|  | 3 | Specify the subject population of interest. | 5 |
|  | 4 | Specify the rater population of interest (if applicable). | 7 |
|  | 5 | Describe what is already known about reliability and  agreement and provide a rationale for the study (if applicable). | 4-5 |
| Methods | 6 | Explain how the sample size was chosen. State the determined number of raters, subjects/objects, and replicate observations. | 5,7,11 |
|  | 7 | Describe the sampling method. | 5-6 |
|  | 8 | Describe the measurement/rating process (e.g. time interval between repeated measurements, availability  of clinical information, blinding). | 7-9 |
|  | 9 | State whether measurements/ratings were conducted independently. | 7 |
|  | 10 | Describe the statistical analysis. | 10-11 |
| Results | 11 | State the actual number of raters and subjects/objects  which were included and the number of replicate observations which were conducted. | 5,7,12 |
|  | 12 | Describe the sample characteristics of raters and  subjects (e.g. training, experience). | 5,7 |
|  | 13 | Report estimates of reliability and agreement including measures of statistical uncertainty. | 12-17 |
| Discussion | 14 | Discuss the practical relevance of results. | 17-21 |
| Auxiliary material | 15 | Provide detailed results if possible (e.g. online). | Appendix |
